# Supplementary material for: An on-demand, drop-on-drop method for studying enzyme catalysis by serial crystallography
Source: Nat Commun. 2021 Jul 22;12:4461. doi: 10.1038/s41467-021-24757-7 (PMC8298390; doi:10.1038/s41467-021-24757-7)
Supplement: Supplementary file 1 — Supplementary information [file 41467_2021_24757_MOESM1_ESM.pdf]

SUPPLEMENTARY INFORMATION

SUPPLEMENTARY NOTES

Supplementary note 1

The main challenge of the mixing techniques is to fundamentally reduce the time that is necessary for diffusion of the ligand into the crystals. Because the average size of an ADE drop (4 nL ≃ 200 µm diameter sphere) is much bigger than the size of microcrystals, gradient-driven diffusion through the solution (~3 s for GlcNAc, estimated from the diffusion coefficient [1]) takes considerably longer than the median catalytic turnover. To allow routine time-resolved investigation of all kinds of enzymatic reactions by probing reaction times on the order of tens of milliseconds, the drop-on-drop method needs to support faster diffusion of substrates into crystal slurries. There are several factors that can help to accelerate the equilibration process. Apart from reducing the droplet size, one can change buffer composition, viscosity and/or temperature. However, many of these factors, all of which impact mixing times, cannot be readily modified due to the requirement to maintain the diffraction quality crystal lattice for SFX experiments. For liquid jet systems, one of the strategies aiding at maximizing mixing efficiency was to introduce active mixing components by incorporating three-dimensional features into jet channels, enabling mixing based on striated rather than laminar liquid flow and opening the potential of reaching mixing times of tens of milliseconds [2].

To evaluate whether droplet collisions can actively reduce equilibration times in the drop-on-drop framework, we experimentally determined fluorescence rise times and compared the measured values with computed data from three-dimensional simulations of diffusion and reaction, and multi-compartmental simulations of mass transfer between colliding drops and reaction (Methods). These simulations represent two extreme situations: entirely diffusion-controlled mixing, and nearly instantaneous complete mixing on contact between the droplets. In our experimental tests we monitored

the fluorescence signal of 4 nL ADE drops containing an aqueous solution of two calcium-sensitive dyes (Fura Red and Fluo-5N, both at 1 mM concentration) after addition of 60 pL droplets of aqueous 100 mM calcium chloride solution. Supplementary Figure 5 shows the exemplary experimental data recorded before and after the addition of the calcium chloride stock solution from PEI. In the presence of calcium ions, the Fura Red emission peak shifts its maximum from 670 nm to 640 nm whereas the Fluo-5N peak appears only upon calcium ion binding at 515 nm. After fitting normalized spectral components to the data, scaling factors can be extracted for various interaction times. Figure 1b shows the rise of the experimental fluorescence of the two calcium ion-bound dye forms as a function of interaction time (scaled by the inverse tape speed, see Methods). The fluorescence intensity of the more sensitive Fura Red dye almost reaches its maximum for the shortest explored mixing times (rise time of 99±21 ms), whereas the fluorescence of the less sensitive Fluo-5N dye rises slower with rise time of 157±34 ms. Fluo-5N fluorescence signal reaches 95% of its maximum value within about 470 ms and the solution is close to equilibrium.

In the reaction-diffusion simulations, three 60 pL droplets were allowed to diffuse into a 4 nL drop containing the same dyes as used experimentally. The geometry is shown in Supplementary Figure 2, and data for the reaction-diffusion scheme are presented in Supplementary Tables 1 and 2. This simulation configuration assumes that the PEI droplets are resting on top of the nanoliter-sized ADE drop, with no initial mixing. When comparing the resulting simulated reaction-diffusion data to the experimental fluorescence rise, it is clear that diffusion alone leads to an almost one order of magnitude slower rise time than is observed (Figure 1b).

The other extreme is very rapid mixing due to hydrodynamic flow, initiated when the droplets are contacted. This is modeled using simulations that include mass flow between several compartments representing the drop volumes (Supplementary Figure 3). The mass flow rate of calcium ions from the small PEI drop was first calculated using the relations described in Supplementary Table 2 and included in these schemes. The simulations (Figure 1b) showed that by including proportional mass flow of calcium between the PEI and ADE droplets, mixing driven by hydrodynamic flow can result in a

homogenous droplet on a sub-millisecond timescale (~0.7 ms), which is much faster than observed. Since the variables used in the simulation are physically based and validated, the fact that neither model agrees with experiment means that they are not an accurate representation of the physics of the system. The mixing process is clearly more complex.

To develop an improved model, we incorporated the results of a study that investigates how aqueous droplets merge in the velocity regime used in this work. That work showed that mixing following collision of one small with one large drop is initially driven by hydrodynamic mass flow and formation of internal jets within the combined drop volume [3]. To evaluate whether the internal jets could account for the fluorescence rise times, we assume that they are formed nearly instantaneously, and are a starting point for simple diffusion and reaction through the remainder of the droplet volume.

The simple three-dimensional drop-on-drop system shown in Supplementary Figure 2 was adapted as shown in Supplementary Figure 4 to include jet structures within the combined drop volume. These internal jets result in partial mixing and reaction as soon as they are formed. We simulated the effect of this partial mixing of fluids on the reaction time of calcium and dyes throughout the combined drop volume. We modeled the jets as a conical PEI shape. This was done by first calculating the Weber number of the large ADE droplet sitting on the tape (Supplementary Table 3) and building an approximate three-dimensional model of the internal jet formed 1 ms after impact as described in literature. Three ~60 pL PEI jets (containing 100 mM calcium chloride) were constructed and were allowed to diffuse into and react with the dye-containing ADE drop (containing 1 mM of both, FuraRed and Fluo-5N dyes). By rearranging the PEI droplets to extend deep into the ADE volume, we found that about half of the total calcium-dye complexes are formed faster than in the simplified model (Supplementary Figure 6). Following the rapid rise, when the calcium concentration decreases and therefore the gradient driven diffusion slows down, the two reactions advance similarly. Overall, the rate of formation of both fluorescent complexes was only moderately increased. This indicates that the droplet mixing process is likely to be a combination of hydrodynamic flow and diffusion, but that the initial model underpredicts the extent of reaction at early times. The difference between the two three-

dimensional geometries (i.e., conical versus flat PEI droplets with otherwise identical reaction schemes) suggests that fluid mixing using the drop-on-drop instrument is greatly sped up by the droplet collision. Future work will focus on gaining an improved description of this process in order to develop predictive models that will enable both design and interpretation of drop-on-drop experiments.

Supplementary note 2

To experimentally test the time necessary for a ligand binding in enzyme active sites within microcrystals using the drop-on-drop system, we first used hen egg white lysozyme (HEWL) as a model system. HEWL is very robust and has been used to demonstrate utility of various sample delivery methods, including proof-of-principle experiments for emerging mixing strategies. Previous mix-and-diffuse studies used the same crystal system and the substrate analogue N,N',N''-triacetyl chitotriose (CTO, MW = 627.6 g/mol) as an active site ligand [4,5]. In our experiments we used 3 × 3 × 5 μm^3 HEWL microcrystals and a monosaccharide, N-acetyl-D-glucosamine (GlcNAc, MW = 221.2 g/mol). GlcNAc is a glucose derivative which, while having a lower affinity for HEWL than CTO (K_D = 47.6 mM vs 13 μM, respectively [6]), has lower molecular weight and thereby is better suited to evaluate sample delivery strategies with relatively fast mixing times. The active site cleft of HEWL is a long groove that can accommodate up to six sugars simultaneously (subsites A-F [7]). In both of our structures (0.6 s and 2 s mixing time), GlcNAc is found in the subsite D held in the active site through a network of hydrogen bonds (Figure 2), which is adjacent to the site where the catalytic cleavage occurs (sites D/E). The position observed in our structures corresponds well to that reported in other studies involving GlcNAc [7-9]. In particular, the orientation of the sugar moiety closely resembles the one reported in Tanley et al. [9] with root-mean-square deviation for all GlcNAc atoms of 0.9 Å, as calculated with DockRMSD [10], with the only difference that in our structures GlcNAc is bound as the α and not β stereoisomer (defined by the conformation of the O1 anomeric oxygen, Figure 2). This is unsurprising given that the isomeric forms can freely interconvert in aqueous solution, with the ratio of the two anomers being context dependent, e.g., influenced by pH or temperature.

Supplementary note 3

CTX-M-15 is a widely distributed enzyme capable of hydrolyzing penicillins and some cephalosporins, but it very poorly hydrolyzes, and can be inhibited by carbapenems, which are often considered 'last resort' β-lactam antibiotics to treat serious infections by multidrug resistant Gram-negative pathogens [11,12]. For our drop-on-drop XFEL studies, we selected ertapenem, a clinically important carbapenem highly soluble in water (MW = 475.52 g/mol). Analysis of the crystal packing of CTX-M-15 crystals indicated that, despite the relatively low solvent content of 42%, the diffusion of a small molecule like ertapenem to the active site should be possible (Supplementary Figure 10). Our kinetic data (Supplementary Table 8) indicate that ertapenem is slowly hydrolyzed by CTX-M-15 (k_{cat} = 0.001 s^{-1}). However, it readily binds to (K_M = 11.6 μM) and acylates the active site serine of the enzyme (onset of acylation, k_2/K = 7344 $M^{-1}s^{-1}$) and has an apparent inhibition constant ($K_{i\ app}$ = 1.8 μM) that is equivalent to the inhibitor relebactam that is approved for clinical use in humans [13]. The acyl-enzyme complex is therefore very slowly hydrolyzed (i.e. the steps outlined in panel b in Supplementary Figure 12 occur very slowly), resulting in the observed slow turnover. Carbapenem hydrolysis by class A SBLs, such as the CTX-M enzymes, is known to result in three possible derived products due to the possibility of tautomerization of the acyl-enzyme through migration of the C2=C3 double bond (Supplementary Figure 12). Indeed, room-temperature serial data collected in a fixed target setup at Diamond Light Source microfocus beamline I24 from microcrystals pre-soaked with ertapenem (10 min) are consistent with the formation of an acyl-enzyme species with the β-lactam ring opened (i.e., covalently bound ertapenem) and no evidence for non-covalently reacted ertapenem (Supplementary Figure 9). In this crystal structure derived from pre-soaked crystals, hydrolyzed ertapenem was modeled as the imine in the Δ^1 tautomer, with the C-2 sidechain in the '*R*' configuration and the sulfur atom out of the plane of the pyrroline ring. The benzoic acid atoms of the R_1 side chain were not defined in the electron density and were removed from the final model. The ertapenem-derived product is stabilized through multiple hydrogen bonds with the protein main chain, including the interaction of the acyl-enzyme C-7 carbonyl oxygen with the

oxyanion hole formed by the backbone amides of Ser237 and Ser70 that is widely conserved in class A β-lactamases [10]. In addition, the ertapenem pyrroline ring forms hydrophobic interactions with Tyr105, a residue associated with ligand interactions in multiple class A enzymes [13]. The C-6-hydroxyethyl group forms hydrogen bonds with Lys73 and Asn132 that orient it to allow interactions of the hydroxyl oxygen with a water molecule that is postulated to be required for deacylation and ultimate resolution of the acyl-enzyme complex [13] (DW in Figure 2f). It has previously been suggested that this interaction deactivates the water molecule and is incompatible with deacylation as outlined in Supplementary Figure 12, preventing turnover and thus inhibiting the enzyme [13]. In accordance with this suggestion, the C-6-hydroxyethyl group is seen in a different position (pointing away from DW) from the one observed here in carbapenem complex structures of class A β-lactamases which more efficiently turn over these substrates (e.g., SFC-1 [14]).

In the 2 s mixing point XFEL structure the ertapenem-derived product is also present as the (*R*)-Δ^1-pyrroline tautomer, though with a 90° rotation of the C-3 carboxylate as well as slight reorientation of the C-6 hydroxyethyl group that positions the hydroxyl oxygen further away from the deacylating water (Figure 2f and Supplementary Figure 9). The whole R_1 side chain was not modeled due to poorly defined density for this region. This contrasts the structure obtained at the 10 min mixing point in which only the benzoic acid atoms of the R_1 side chain could not be modeled (Supplementary Figure 9), suggesting the ligand may be stabilized over time. The poor electron density for ertapenem (as compared to GlcNAc in the HEWL complexes) is likely due to the possibility of multiple ertapenem-derived products forming in the CTX-M-15 active site, in contrast to the non-enzymatic binding of GlcNAc to HEWL. Consistently, occupancies were refined to 0.74 and 0.84 for the 2 s drop-on-drop structure and 10 min fixed target structures, respectively. Additional XFEL studies are required to enable us to more clearly define the tautomeric state of the pyrroline ring and the geometry of the C6-hydroxyethyl group during hydrolysis, and to validate that the complexes modeled to our SFX data are relevant to catalysis in solution and/or to inhibition.

SUPPLEMENTARY FIGURES

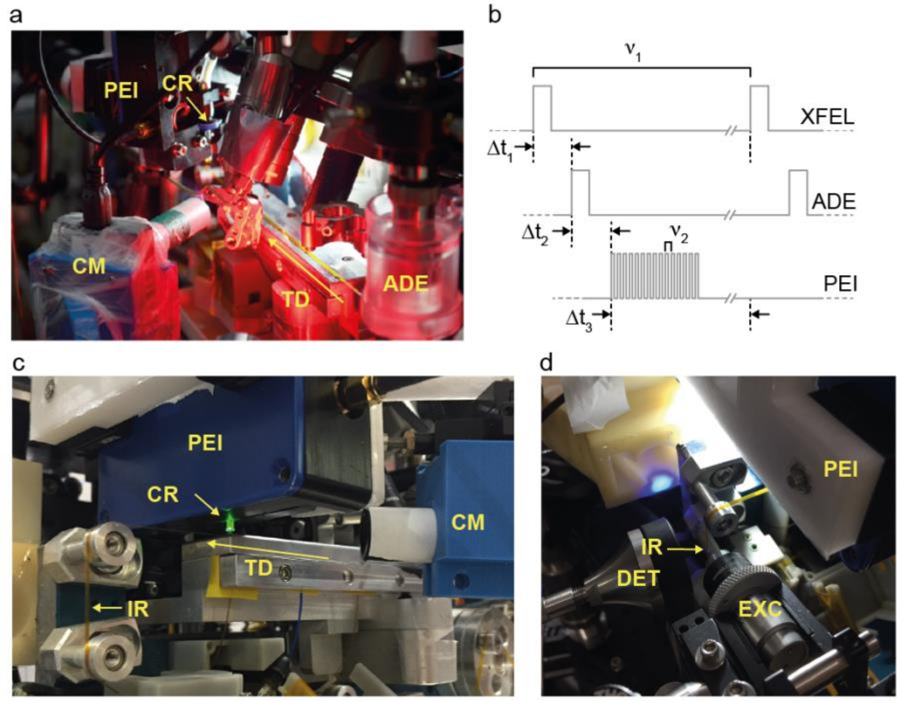

Supplementary Figure 1. **Drop-on-drop experimental setup**. **a** Experimental setup during the XFEL experiments at SACLA. **b** Schematic representation of the pulse sequence timing. The acoustic droplet ejection (ADE) of the microcrystal slurry is triggered by a pulse delayed relative to the 30 Hz (ν_1) XFEL master clock (Δt_1). Another TTL pulse synchronized with the ADE with an additional time delay (Δt_2) is sent to the piezoelectric injection (PEI), which adds ligand solution in discrete picoliter-sized droplets at various frequency (here, 0.7 – 6.1 kHz, ν_2) across the slurry drop. Reaction time is defined as the time between droplet merging and the moment in which they arrive at the XFEL beam position (Δt_3). **c** and **d** Experimental setup during the fluorescence measurements at LBNL. In panels **a**, **c** and **d**: PEI - piezoelectric injector; CR - cartridge; ADE - acoustic droplet ejector; CM - calibration camera, IR - interaction region; TD - tape direction; EXC - incoming excitation light passing through focusing optics; DET - optics for collecting the emitted fluorescence signal.

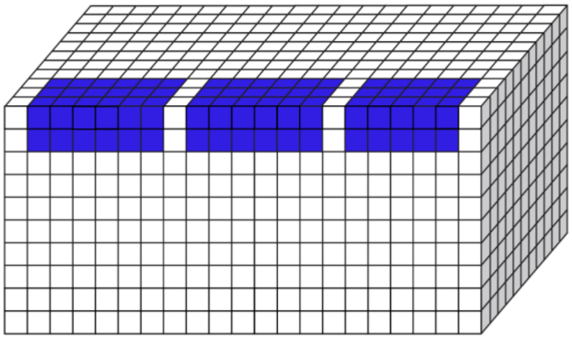

Supplementary Figure 2. **Cross sectional representation of the three-dimensional reaction-diffusion system built to investigate the competition between hydrodynamic mass flow and simple diffusion.** The system is supported on one side, and all volumes are presented as cubes with 1000 μm^3 volume, with a total of 4200 compartments. The choice of Cartesian coordinates rather than spherical polar coordinates simplifies the simulations and does not significantly influence their predictions in this study. A cut through the full volume is shown. White compartments correspond to the acoustic droplet ejector (ADE) droplet (which contains 1 mM of both dyes: Fluo-5N and Fura Red), and the blue compartments correspond to the three piezoelectric injector (PEI) droplets (which contain 100 mM of Ca(II) solution each). All remaining layers are either identical or contain only ADE (white) compartments. Note that one PEI droplet is smaller in volume than the remaining two droplets (i.e., 50 compartments) to ensure that all PEI droplets are surrounded by ADE compartments in all five directions.

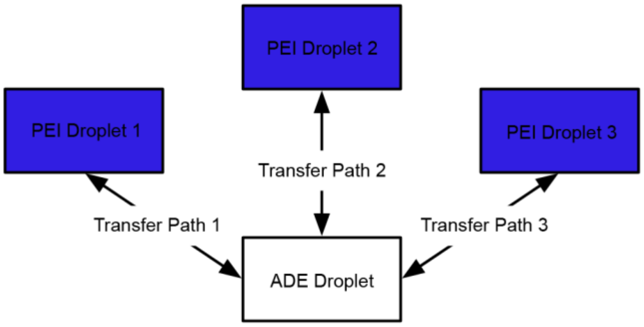

Supplementary Figure 3. **Schematic representation of the compartmental system constructed using Kinetiscope to model mass transfer in the drop-on-drop setup.** The system consists of four compartments representing droplets containing the dyes (ADE) and calcium (PEI). All volumes and concentrations match those used in the reaction-diffusion system (4 nL ADE droplets and approximately 60 pL PEI droplets). Transfer paths are bidirectional and correspond to the proportional flow of calcium into and out of the compartments. Transfer rates are calculated according to relations detailed in Supplementary Table 2.

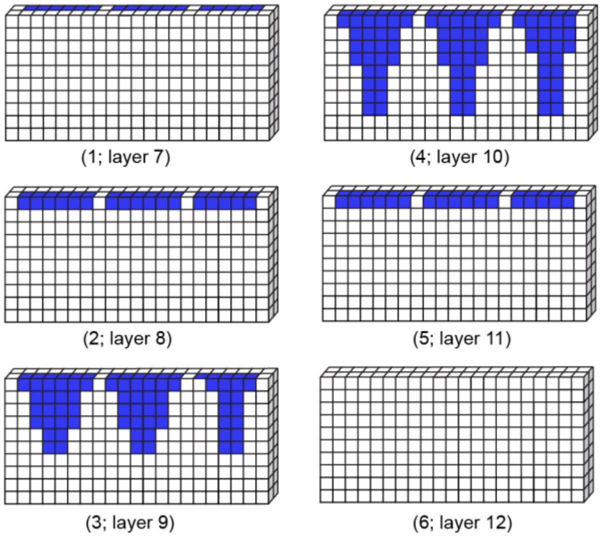

Supplementary Figure 4. **Collection of cross-sectional images of the conical PEI drops built to improve the model shown in Supplementary Figure 2.** The images are arranged sequentially, starting from layer 7 through layer 12. All physical and kinetic parameters are identical to those used in the alternative three-dimensional simulation (Methods, Supplementary Figure 2, Supplementary Tables 1 and 2). The white compartments represent the 4 nL acoustic droplet ejector (ADE) droplet (which contains 1 mM of both calcium-sensitive dyes), while the blue compartments represent the 60 pL piezoelectric injector (PEI) droplets (all of which contain 100 mM of calcium(II) solution). The Weber number of the ADE droplet was calculated to equal 2.51, and conical jets were modeled after experimental observations of jets formed in droplets with similar Weber number values (Supplementary Table 3).

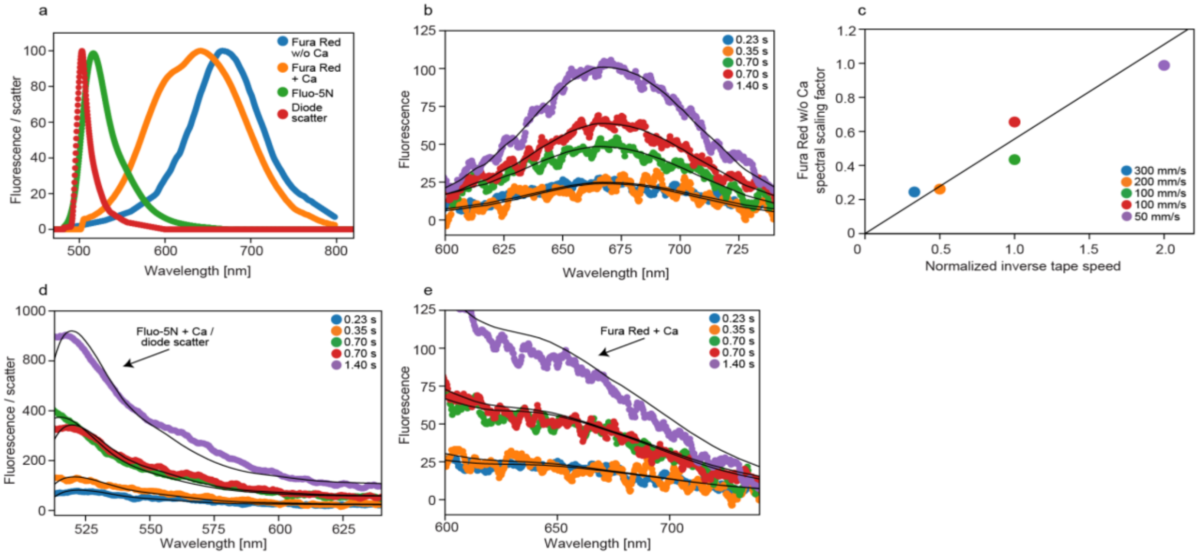

Supplementary Figure 5. **Details of the fluorescence data evaluation. a** Spectra of fit components. The spectrum of Fluo-5N was used as given by the provider (Thermo Fisher) while the others were obtained as described in the Methods section. **b** Fluorescence of droplets of 1 mM Fura Red and Fluo-5N without the addition of calcium recorded at different tape speeds. Fitted curves are shown in black. **c** The scaling factors corresponding to the fit component in **b** of the Fura Red species without calcium plotted versus the inverse tape speed. The points lie on a line as expected for the different duration of stay within the probed volume. This rationalizes the scaling with the inverse tape speed as presented in Figure 1. **d** and **e** Fluorescence of droplets of 1 mM Fura Red and Fluo-5N with the addition of calcium containing droplets recorded after the indicated incubation time. Each measurement represents the average of four scans integrated over 300 ms and was repeated between 10 and 50 times. The main components of each spectral region are indicated, and the fit results are shown in black. The fitted scaling factors of the Fura Red and Fluo-5N spectral components are multiplied by the normalized inverse tape speed and presented and discussed in Figure 1.

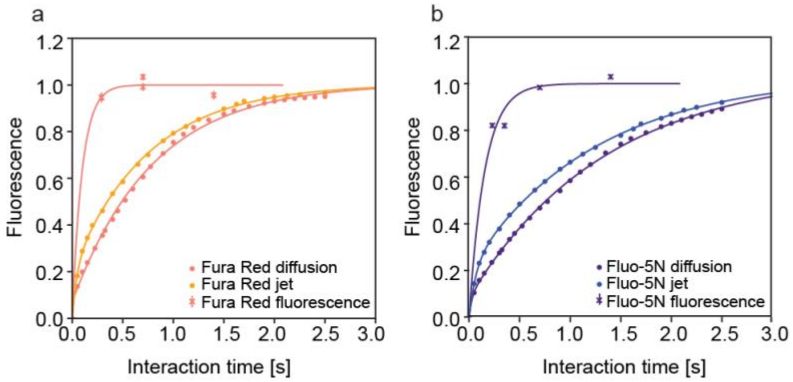

Supplementary Figure 6. **Calcium-dye complex formation in diffusion simulations with different starting conditions and measured fluorescence.** The simulations of the diffusion and binding of calcium to Fura Red (**a**) and Fluo-5N (**b**) dyes start from resting PEI droplets on top of the larger ADE droplet (diffusion, Supplementary Figure 2) or from the PEI droplets penetrating the ADE droplet through internal jets (jet, Supplementary Figure 4). For comparison, the measured fluorescence obtained from the scaling of the normalized spectral components (crosses) and their errors of the fits are also shown. Solid lines correspond to fitted exponential functions and are used in the simulated curves for normalization.

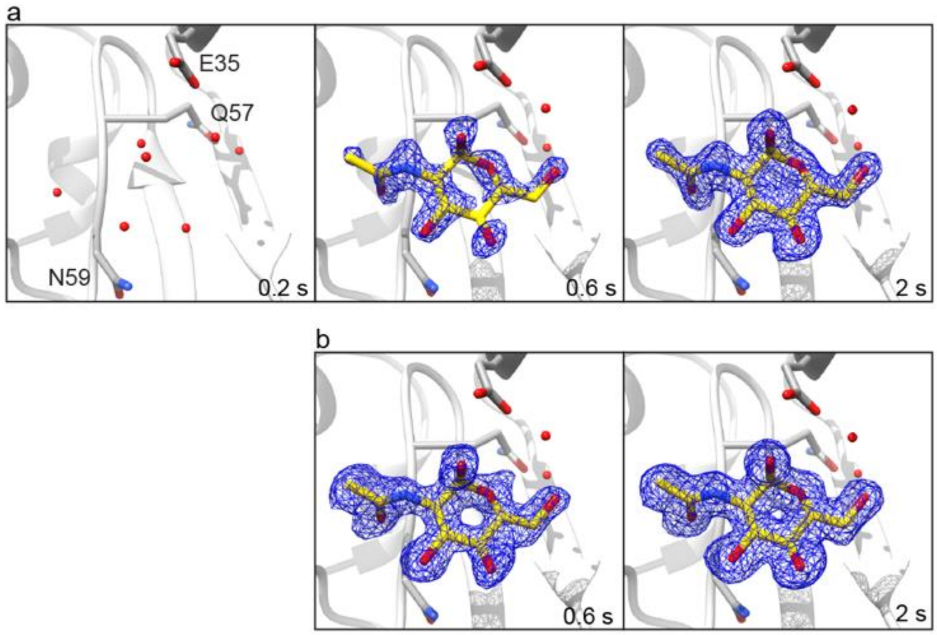

Supplementary Figure 7. **Close-up views of the lysozyme active site after mixing with N-acetyl-D-glucosamine. a** $F_{o\ (\text{time point})} - F_{o\ (\text{resting})}$ isomorphous difference electron density maps displayed at $\pm 3.5\sigma$ and carved 1.5 Å around the ligand site are displayed in blue (positive) and orange (negative). The model used for calculating the maps (resting state structure) did not include the GlcNAc molecule, which is shown here only for reference. **b** $mF_o - DF_c$ polder OMIT difference density map contoured at $\pm 3\sigma$ and carved 1.5 Å around the ligand site is displayed in blue (positive) and orange (negative). The GlcNAc molecule from the 2 s time point structure was used to carve the 200 ms time point map. All maps are at 1.45 Å resolution. Graphic was created using the UCSF Chimera package [15].

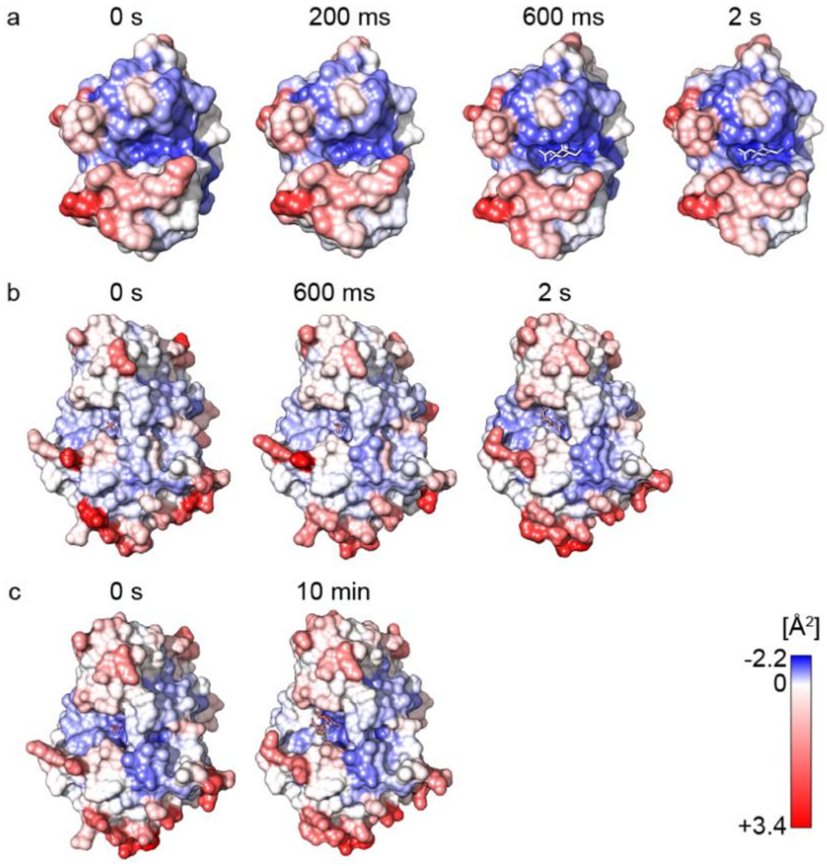

Supplementary Figure 8. **B-factor comparison of HEWL and CTX-M-15 data reveals no notable difference in the B-factor profiles.** Normalized B-factors [16] are displayed per residue for the XFEL HEWL (**a**), XFEL CTX-M-15 (**b**) and synchrotron CTX-M-15 (**c**) structures. Scale bar corresponds to all panels. Graphic was created using the UCSF Chimera package [15].

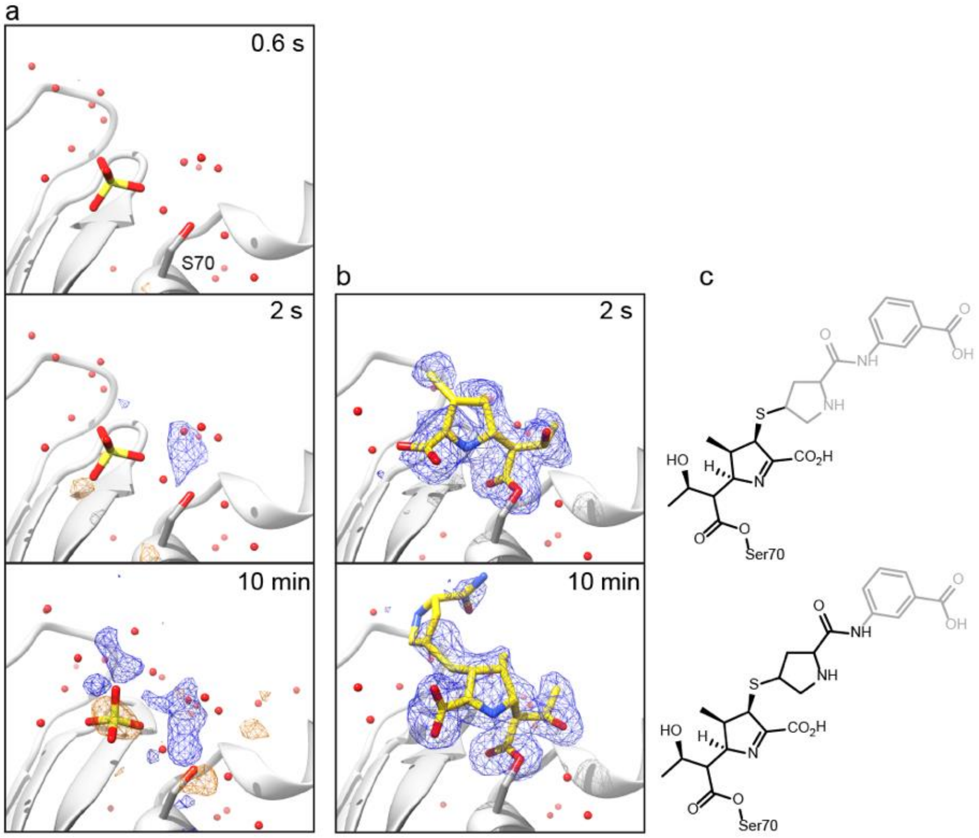

Supplementary Figure 9. **Close-up views of the CTX-M-15 active site after mixing with the substrate ertapenem reveal the acyl-enzyme intermediate at 2 s and 10 minutes. a** $F_{\mathrm{o\ (time\ point)}} - F_{\mathrm{o\ (resting)}}$ isomorphous difference electron density maps displayed at ±3σ in blue (positive) and orange (negative) with the respective resting state structures. The maps were calculated to 1.6 Å (0.6 s and 2 s time points, XFEL data) or 1.65 Å (10 min time point, synchrotron data) resolution and carved 1.5 Å around the ertapenem and sulphate binding site. The main negative electron density map peak can be interpreted as a concurrent disappearance of the sulphate ion, which is bound at the active site in the resting state structure. **b** $mF_o - DF_c$ polder OMIT difference electron density map contoured at ±3σ and carved 1.5 Å around the ligand site is displayed in blue for 2 s (1.55 Å) and 10 min (1.65 Å) data sets. **c** Chemical structures of ring-opened adducts modeled in the 2 s and 10 min datasets. Greyed atoms represent parts of the molecules that were omitted from the model. Graphic was created using the UCSF Chimera package [15].

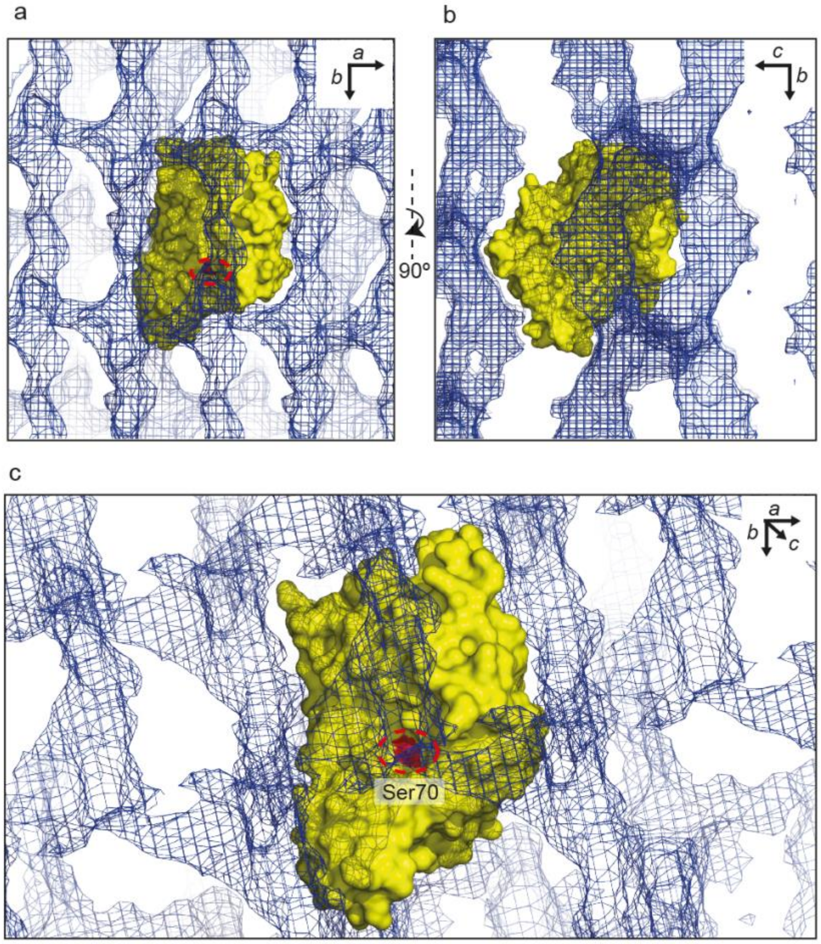

Supplementary Figure 10. **Solvent channels in CTX-M-15 crystals.** The solvent channel maps are contoured at 2.5 Å, meaning that a spherical object of 2.5 Å radius can be placed anywhere in the map without steric clash with CTX-M-15. **a** The main crystal solvent channel is extending along the *b* axis with a radius of 7.8 Å and bottlenecks in the *c*, *a*/*b* and *b* directions of 2.4, 3.8 and 5 Å, respectively. **b** 90º rotation from **a**. **c** Close-up active site view. Ser70 is highlighted in red. The solvent channel maps have been generated with MAP_CHANNELS [17]. This figure was created using PyMOL [18].

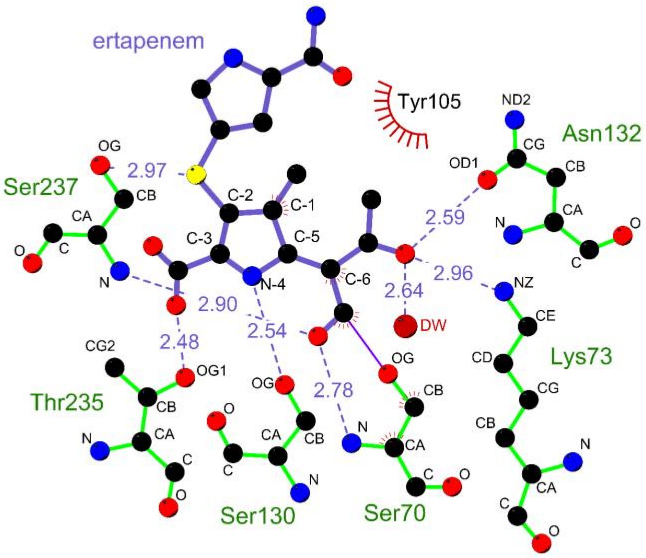

Supplementary Figure 11. **Structure of ring-opened adduct of ertapenem bound to CTX-M-15.** Ertapenem hydrogen bonding network (blue dotted lines) based on the 10 min soak synchrotron structure. Residues involved in hydrophobic contacts are shown as a curved comb. Deacylating water is represented by a red sphere (DW). The figure was generated with Ligplot [19].

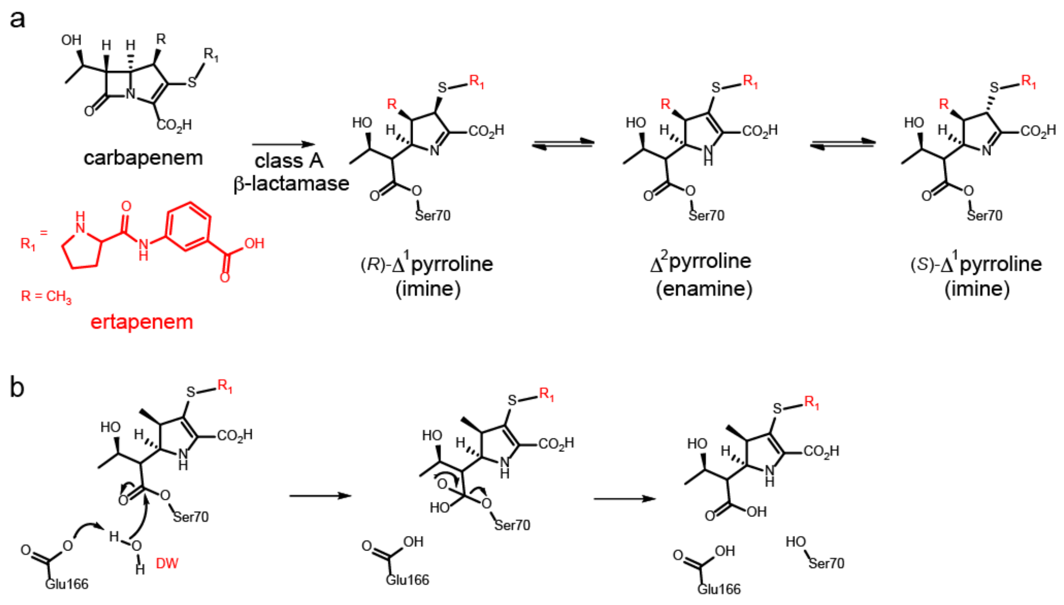

Supplementary Figure 12. **Schematics of the mechanism of ertapenem hydrolysis by CTX-M-15. a** Acyl-enzyme intermediates formed after reaction of carbapenems with class A β-lactamases. Carbapenem degradation by class A β-lactamases results in the formation of three possible major acyl-enzyme products that can tautomerize at the active site. **b** The deacylation mechanism of the ertapenem CTX-M-15 acyl-enzyme. Left, Glu166 acts as a general base, activating the deacylating water (DW) to attack the acyl-enzyme carbonyl. This forms an unstable tetrahedral transition state (middle) that quickly resolves to liberate the hydrolyzed ertapenem-derived product (right). For clarity, only the Δ^2-pyrroline isomer of ertapenem is shown in panel b. Note, the liberated product can also tautomerize between Δ^1- and Δ^2-pyrroline forms in solution. Figure based on Reference 20 and 21.

SUPPLEMENTARY TABLES

Supplementary Table 1. **Parameters of three-dimensional kinetics simulation model.**

Droplet source	Total number of compartments [a]	Droplet dimensions (length × width × height; μm^3)	Final volume (nL)
ADE	4030	210 × 200 × 100	4
PEI	170 [b]	60 × 50 × 20 [c]	~0.060 [c]

[a] All volume elements are represented as cubes, with sides that are 10 × 10 × 10 μm^3.

[b] Three PEI droplets were included in the simulation model. This value corresponds to the combined number of compartments of all three droplets.

[c] These values correspond to a single PEI droplet.

Supplementary Table 2. **Reaction scheme parameters used in kinetics simulations.**

Reactant	Molecular weight ($g \cdot mol^{-1}$)	Concentration (mM)	Ca(II) dissociation constant (K_D; nM)	k_{on} ($s^{-1} \cdot M^{-1}$)	k_{off} (s^{-1}) [a]	Diffusion coefficient ($\mu m^2 \cdot s^{-1}$) [b]
Calcium(II)	40.08 [c]	100	N/A	N/A	N/A	793 [d]
Fura Red	1088.99 [e]	1	$9 \cdot 10^4$ [e]	$5 \cdot 10^8$ [f]	200	300 [g]
Fluo-5N	1127.92 [e]	1	400 [e]	$3 \cdot 10^8$ [h]	$2.7 \cdot 10^4$	140 [h]

[a] Calculated as $K_D \cdot k_{on}$.

[b] Reaction-diffusion kinetics simulations assume that the diffusion coefficient of the uncomplexed dye is equal to that of the calcium-dye complex.

[c] PubChem Compound Summary for CID 5460341, Calcium. PubChem, National Center for Biotechnology Information. https://pubchem.ncbi.nlm.nih.gov/compound/Calcium (2020).

[d] Parkhurst, D.L. and Appelo, C.A.J., Description of Input for PHREEQC Version 3—A Computer Program for Speciation, Batch-Reaction, One-Dimensional Transport, and Inverse Geochemical Calculations U.S. Geological Survey Open-File Report. https://www.aqion.de/site/194 (2011).

[e] Provided by the supplier.

[f] The association constant of Fura-2 dye to calcium was used [22].

[g] The diffusion coefficient of Mag-Fura Red dye was used [23].

[h] The diffusion coefficient and association constant of Fluo-5F to calcium were used [24].

Supplementary Table 3. **Droplet mixing parameters calculated for use in compartmental simulations controlled purely by hydrodynamic flow with no diffusive movement of species.**

Parameter	Value	Notes
droplet radius (m)	$1{\cdot}10^{-4}$	For the complete engulfment of the PEI droplet by the ADE droplet, use the ADE droplet radius.
characteristic travel length *l* (m)	$2{\cdot}10^{-4}$	Defined as droplet diameter.
relative velocity *v* ($m{\cdot}s^{-1}$)	$95{\cdot}10^{-2}$	Estimated from PEI ejection and tape speeds.
transfer rate *t* (s^{-1})	$4.75{\cdot}10^{3}$	Defined by Supplementary Equation 1: $$t = \frac{1}{\tau_{\mathrm{Conv}}} \quad (1)$$ where τ_{Conv} is convection time [25].
Weber number We (unitless)	2.51	Defined by Supplementary Equation 2: $$\mathrm{We} = \frac{\rho v^2 l}{\sigma} \quad (2)$$ where water density ρ and surface tension σ values were taken from literature [26, 27].

Supplementary Table 4. **Drop-on-drop, time-resolved SFX data collection parameters.**

Protein	CTX-M-15			Lysozyme			
ADE drop volume [nL]	3	3	3	3	3	3	3
Ligand	-	Ertapenem	Ertapenem	-	GlcNAc	GlcNAc	GlcNAc
PEI droplet volume [nL]	-	0.12	0.12	-	0.12	0.12	0.12
PEI droplet burst frequency [kHz]	-	2	0.7	-	6.1	1	1
No. PEI droplets per burst	-	20	20	-	20	10	10
Theoretical number of PEI droplets intersecting with a 3 nL ADE drop	-	4	5	-	4	2	6
Belt speed [$mm \cdot s^{-1}$]	100	100	30	300	300	100	30
Enzyme-ligand reaction time [ms]	-	667	2000	-	200	667	2000
Protein concentration in the crystal [mM] [a]	29	29	29	58	58	58	58
Protein concentration in the drop [mM]	0.28	0.28	0.28	1.75	1.75	1.75	1.75
Ligand stock concentration [mM]	-	800	800	-	226	226	226
Final theoretical ligand concentration [mM]	-	110.3	133.3	-	31.2	16.7	43.7
Ligand consumption [$\mu L \cdot min^{-1}$]	-	4.3	4.3	-	4.3	2.16	2.16
Total ligand consumption for tr-SFX dataset [µL]	-	258	215	-	155	181	84
Total ligand consumption for tr-SFX dataset [µmol]	-	206	172	-	35	41	19
Total crystal slurry consumption for tr-SFX per dataset [µL]	300	420	275	216	162	378	176
Total protein consumption for tr-SFX dataset [mg]	2.4	3.4	2.2	16.2	12.5	9.5	4.4

[a] Holton J., CCP4BB Archive, https://www.jiscmail.ac.uk/cgi-bin/wa-jisc.exe?A2=ind1012&L=CCP4BB&D=0&O=D&P=111998, 2010

Supplementary Table 5. **Data collection and refinement statistics for HEWL structures.**

	7BHK<br>resting, drop-on-drop	7BHL<br>0.2 s, drop-on-drop	7BHM<br>0.6 s, drop-on-drop	7BHN<br>2 s, drop-on-drop
**Data collection**				
No. of collected images	68,844	47,394	101,329	67,257
No. of hits [a]	36,676	17,563	18,700	9,611
No. of indexed images [b]	6,509	4,588	5,630	6,779
No. of integrated lattices [b]	8,833	6,253	7,690	10,288
No. of merged lattices [b]	8,735	6,202	7,590	10,201
Space group	$P4_32_12$	$P4_32_12$	$P4_32_12$	$P4_32_12$
Unit cell parameters (Å)				
a, *b*, *c* (Å)	78.8, 78.8, 38.0	78.8, 78.8, 38.0	78.8, 78.8, 38.0	78.8, 78.8, 38.0
α, β, γ (°)	90, 90, 90	90, 90, 90	90, 90, 90	90, 90, 90
No. unique reflections	21782 (1056)	21782 (1056)	21782 (1056)	21782 (1056)
Resolution (Å)	55.72-1.45 (1.48-1.45)	55.72-1.45 (1.48-1.45)	55.72-1.45 (1.48-1.45)	55.72-1.45 (1.48-1.45)
<I>	18423 (241)	19464 (245)	18671 (266)	18509 (238)
$\langle I/\sigma(I)\rangle$	63.741 (0.583)	55.750 (0.497)	57.592 (0.601)	68.773 (0.648)
Completeness (%)	100 (100)	100 (100)	100 (100)	100 (100)
Multiplicity	80.15 (17.08)	52.84 (11.59)	67.39 (13.99)	125.38 (24.65)
R_{split} (%)	18.7 (130.0)	20.1 (129.4)	19.7 (133.5)	13.6 (122.8)
$CC_{1/2}$ (%)	95.2 (5.4)	94.4 (3.3)	94.9 (0.3)	97.8 (3.4)
Wilson B value (Å^2) [c]	20.4	19.8	20.1	20.4
**Refinement**				
Resolution range	34.23-1.45 (1.50-1.45)	34.23-1.45 (1.50-1.45)	34.23-1.45 (1.50 -1.45)	34.23-1.45 (1.50-1.45)
No. reflections	21704	21663	21699	21738
R_{work}	0.1944 (0.4572)	0.1970 (0.4746)	0.1923 (0.4572)	0.1812 (0.4357)
R_{free}	0.2144 (0.4257)	0.2112 (0.4249)	0.2204 (0.4508)	0.1941 (0.4020)
No. of non-hydrogen atoms				
Protein	1040	1050	1054	1049
GlcNAc	NA	NA	15	15
Solvent	87	86	88	83
Average B-factors (Å^2)				
Protein	27.03	27.40	26.77	26.27
GlcNAc	NA	NA	29.25	22.62
Solvent	38.39	38.51	39.10	38.79
Ramachandran (%)				
Favored (%)	99	99	99	99
Allowed (%)	1	1	1	1
Outliers (%)	0	0	0	0
R.m.s. deviations				
Bond lengths (Å)	0.005	0.005	0.005	0.005
Bond angles (°)	0.78	0.76	0.80	0.79

Values in parentheses are for the highest resolution shell. [a] from Cheetah [28]; those images with more than 20 reflections. [b] from cctbx.xfel [29]. [c] from Xtriage [30].

Supplementary Table 6. **Data collection and refinement statistics for CTX-M-15 structures.**

	7BH3<br>resting, drop-on-drop	7BH4<br>0.6 s, drop-on-drop	7BH5<br>2 s, drop-on-drop	7BH6<br>resting, fixed target	7BH7<br>10 min, fixed target
**Data collection**					
No. of collected images	59,137	63,778	70,040	76,637	76,800
No. of hits [a]	5,509	22,417	17,469	NA	NA
No. of indexed images [b]	3,051	7,415	8,637	15,843	13,100
No. of integrated lattices [b]	4,794	16,818	18,001	18,661	15,654
No. of merged lattices [b]	4,502	15,151	16,028	17,628	15,207
Space group	$P2_12_12_1$	$P2_12_12_1$	$P2_12_12_1$	$P2_12_12_1$	$P2_12_12_1$
Unit cell parameters (Å)					
a, *b*, *c* (Å)	44.89, 45.60, 117.57	44.93, 45.61, 117.67	44.85, 45.49, 117.56	45.28, 45.96, 118.49	45.26, 45.98, 119.03
α, β, γ (°)	90, 90, 90	90, 90, 90	90, 90, 90	90, 90, 90	90, 90, 90
No. unique reflections	32656 (1585)	35926 (1756)	35730 (1751)	30552 (1491)	30690 (1492)
Resolution (Å)	58.79-1.60 (1.63-1.60)	58.83-1.55 (1.58-1.55)	58.78-1.55 (1.58-1.55)	59.24-1.65 (1.68-1.65)	59.52-1.65 (1.68-1.65)
$\langle I \rangle$	26161 (631)	24770 (675)	24082 (512)	29215 (3402)	30765 (3356)
$\langle I/\sigma(I) \rangle$	18.484 (0.45)	36.063 (0.86)	39.566 (0.71)	47.659 (6.38)	53.658 (6.40)
Completeness (%)	100 (100)	100 (100)	100 (100)	100 (100)	100 (100)
Multiplicity	26.45 (15.16)	114.15 (43.49)	134.23 (48.45)	61.27 (11.15)	47.09 (9.20)
R_{split} (%)	44.1 (137.8)	23.7 (116.3)	21.0 (123.2)	25.4 (48.8)	24.4 (58.3)
$CC_{1/2}$ (%)	83.3 (2.2)	94.9 (10.3)	96.1 (9.7)	89.3 (56.9)	90.2 (25.8)
Wilson B value (Å^2) [c]	17.5	18.4	19.3	13.6	14.0
**Refinement**					
Resolution range	31.99-1.60 (1.66-1.60)	32.01-1.55 (1.61-1.55)	31.94-1.55 (1.61-1.55)	42.85-1.65 (1.71-1.65)	59.52-1.65 (1.71-1.65)
No. reflections	32362 (2938)	35838 (3461)	35617 (3407)	30548 (2977)	30691 (3009)
R_{work}	24.60 (43.6)	17.89 (41.58)	17.78 (41.85)	0.1630 (0.2068)	0.1618 (0.2188)
R_{free}	29.64 (49.52)	20.60 (47.53)	20.20 (43.25)	0.1967 (0.2458)	0.1934 (0.2472)
No. of non-hydrogen atoms					
Protein	2005	1972	1979	1998	1968
Ertapenem	NA	NA	16	NA	24
Solvent	151	164	145	229	206
Average B-factors (Å^2)					
Protein	29.26	28.83	26.80	16.92	17.60
Ertapenem	NA	NA	38.64	NA	35.68
Solvent	38.11	40.68	37.54	31.89	32.30
Ramachandran (%)					
Favored	98.44	98.44	98.44	98.44	98.44
Allowed	1.17	1.17	1.17	1.17	1.17
Outliers	0.39	0.39	0.39	0.39	0.39
R.m.s deviations					
Bond lengths (Å)	0.011	0.010	0.010	0.009	0.008
Bond angles (°)	1.124	1.028	1.02	1.03	1.01

Values in parentheses are for the highest resolution shell. [a] from Cheetah [28], those images with more than 20 reflections. [b] from DIALS [29] or cctbx.xfel [29]. [c] from Xtriage [30].

Supplementary Table 7. **Averaged normalized B-factors ($Å^2$) of residues forming the active site.**

	HEWL [a]	CTX-M-15 [b]
Resting (SACLA)	-0.54	-0.40
0.2 s (SACLA)	-0.53	N/A
0.6 s (SACLA)	-0.63	-0.37
2.0 s (SACLA)	-0.70	-0.40
Resting (DLS I24)	N/A	-0.54
10 min (DLS I24)	N/A	-0.38

[a] Glu35, Asn46, Asp52, Gln57, Ile58, Asn59, Trp63, S91, Ala107, Trp108, Val109.

[b] Ser70, Lys73, Ser130, Asn132, Glu166, Asn170, Ser237, Thr235.

Supplementary Table 8. **Solution steady-state kinetic parameters for ertapenem hydrolysis by CTX-M-15.** Note, ertapenem is poorly hydrolyzed by CTX-M-15. See Supplementary Note 3 for discussion.

k_{cat} (s^{-1})	K_M (μM)	K_{cat}/K_M ($\mu M^{-1}\cdot s^{-1}$)	$K_{i\ app}$ (μM)	k_2/K ($M^{-1}\cdot s^{-1}$)
0.00094 (0.0000075)	11.6 (3.4)	0.000081	1.8	7344 (820)

Standard error in parenthesis, n=3

SUPPLEMENTARY REFERENCES

1 Zhong, Y., Bauer, B. A. & Patel, S. Solvation properties of N-acetyl-β-glucosamine: molecular dynamics study incorporating electrostatic polarization. *J Comput Chem* **32**, 3339-3353, doi:10.1002/jcc.21873 (2011).

2 Knoska, J. *et al.* Ultracompact 3D microfluidics for time-resolved structural biology. *Nat Commun* **11**, 657, doi:10.1038/s41467-020-14434-6 (2020).

3 Tang, C. L., Zhao, J. Q., Zhang, P., Law, C. K. & Huang, Z. H. Dynamics of internal jets in the merging of two droplets of unequal sizes. *J Fluid Mech* **795**, 671-689, doi:10.1017/jfm.2016.218 (2016).

4 Beyerlein, K. R. *et al.* Mix-and-diffuse serial synchrotron crystallography. *IUCrJ* **4**, 769-777, doi:10.1107/S2052252517013124 (2017).

5 Mehrabi, P. *et al.* Liquid application method for time-resolved analyses by serial synchrotron crystallography. *Nat Methods* **16**, 979-982, doi:10.1038/s41592-019-0553-1 (2019).

6 Kumagai, I., Sunada, F., Takeda, S. & Miura, K. Redesign of the substrate-binding site of hen egg white lysozyme based on the molecular evolution of C-type lysozymes. *J Biol Chem* **267**, 4608-4612 (1992).

7 Von Dreele, R. B. Binding of N-acetylglucosamine to chicken egg lysozyme: a powder diffraction study. *Acta Crystallogr D Biol Crystallogr* **57**, 1836-1842, doi:10.1107/s0907444901015748 (2001).

8 Tanley, S. W. M., Schreurs, A. M. M., Helliwell, J. R. & Kroon-Batenburg, L. M. J. Experience with exchange and archiving of raw data: comparison of data from two diffractometers and four software packages on a series of lysozyme crystals. *J Appl Crystallogr* **46**, 108-119, doi:10.1107/S0021889812044172 (2013).

9 Tanley, S. W. M. *et al.* Structural studies of the effect that dimethyl sulfoxide (DMSO) has on cisplatin and carboplatin binding to histidine in a protein. *Acta Crystallogr D* **68**, 601-612, doi:10.1107/S0907444912006907 (2012).

10 Bell, E. W. & Zhang, Y. DockRMSD: an open-source tool for atom mapping and RMSD calculation of symmetric molecules through graph isomorphism. *J Cheminform* **11**, 40, doi:10.1186/s13321-019-0362-7 (2019).

11 Papp-Wallace, K. M., Endimiani, A., Taracila, M. A. & Bonomo, R. A. Carbapenems: past, present, and future. *Antimicrob Agents Chemother* **55**, 4943-4960, doi:10.1128/AAC.00296-11 (2011).

12 Schneider, I. *et al.* New Variant of CTX-M-Type Extended-Spectrum β-Lactamases, CTX-M-71, with a Gly238Cys Substitution in a Klebsiella pneumoniae Isolate from Bulgaria. *Antimicrob Agents Ch* **53**, 4518-4521, doi:10.1128/Aac.00461-09 (2009).

13 Tooke, C. L. *et al.* β-Lactamases and β-Lactamase Inhibitors in the 21st Century. *J Mol Biol* **431**, 3472-3500, doi:10.1016/j.jmb.2019.04.002 (2019).

14 Fonseca, F. *et al.* The basis for carbapenem hydrolysis by class A β-lactamases: a combined investigation using crystallography and simulations. *J Am Chem Soc* **134**, 18275-18285, doi:10.1021/ja304460j (2012).

15 Pettersen, E. F. *et al.* UCSF Chimera--a visualization system for exploratory research and analysis. *J Comput Chem* **25**, 1605-1612, doi:10.1002/jcc.20084 (2004).

16 Parthasarathy, S. & Murthy, M. R. Analysis of temperature factor distribution in high-resolution protein structures. *Protein Sci* **6**, 2561-2567, doi:10.1002/pro.5560061208 (1997).

17 Juers, D. H. & Ruffin, J. MAP_CHANNELS: a computation tool to aid in the visualization and characterization of solvent channels in macromolecular crystals. *J Appl Crystallogr* **47**, 2105-2108, doi:10.1107/S160057671402281X (2014).

18 The PyMOL Molecular Graphics System, Version 2.0 Schrödinger, LLC.

19 Wallace, A. C., Laskowski, R. A. & Thornton, J. M. LIGPLOT: a program to generate schematic

diagrams of protein-ligand interactions. *Protein Eng* **8**, 127-134, doi:10.1093/protein/8.2.127 (1995).

20 Nukaga, M. *et al.* Inhibition of class A β-lactamases by carbapenems: crystallographic observation of two conformations of meropenem in SHV-1. *J Am Chem Soc* **130**, 12656-12662, doi:10.1021/ja7111146 (2008).

21 Lohans, C. T. *et al.* Mechanistic Insights into β-Lactamase-Catalysed Carbapenem Degradation Through Product Characterisation. *Sci Rep* **9**, 13608, doi:10.1038/s41598-019-49264-0 (2019).

22 Delvendahl, I. *et al.* Reduced endogenous Ca^{2+} buffering speeds active zone Ca^{2+} signaling. *Proc Natl Acad Sci U S A* **112**, E3075-3084, doi:10.1073/pnas.1508419112 (2015).

23 Jackson, A. P., Timmerman, M. P., Bagshaw, C. R. & Ashley, C. C. The kinetics of calcium binding to fura-2 and indo-1. *FEBS Lett* **216**, 35-39, doi:10.1016/0014-5793(87)80752-4 (1987).

24 Mironov, S. L. Theory and experiment reveal unexpected calcium profiles in one-dimensional systems. *arXiv:1304.7905v1* (2013).

25 Carroll, B. & Hidrovo, C. Experimental Investigation of Inertial Mixing in Colliding Droplets. *Heat Transfer Eng* **34**, 120-130, doi:10.1080/01457632.2013.703087 (2013).

26 Tanaka, M., Girard, G., Davis, R., Peuto, A. & Bignell, N. Recommended table for the density of water between 0° C and 40° C based on recent experimental reports. *Metrologia* **38**, 301-309, doi:10.1088/0026-1394/38/4/3 (2001).

27 Hauner, I. M., Deblais, A., Beattie, J. K., Kellay, H. & Bonn, D. The Dynamic Surface Tension of Water. *The Journal of Physical Chemistry Letters* **8**, 1599-1603, doi:10.1021/acs.jpclett.7b00267 (2017).

28 Barty, A. *et al.* Cheetah: software for high-throughput reduction and analysis of serial femtosecond X-ray diffraction data. *J Appl Crystallogr* **47**, 1118-1131, doi:10.1107/S1600576714007626 (2014).

29 Winter, G. *et al.* DIALS: implementation and evaluation of a new integration package. *Acta Crystallogr D Struct Biol* 74, 85-97, doi:10.1107/S2059798317017235 (2018).

30 Adams, P. D. *et al.* PHENIX: building new software for automated crystallographic structure determination. *Acta Crystallogr D Biol Crystallogr* 58, 1948-1954, doi:10.1107/s0907444902016657 (2002).
