# Supplementary material for: An on-demand, drop-on-drop method for studying enzyme catalysis by serial crystallography
Source: Nat Commun. 2021 Jul 22;12:4461. doi: 10.1038/s41467-021-24757-7 (PMC8298390; doi:10.1038/s41467-021-24757-7)
Supplement: Supplementary file 3 — Description of Additional Supplementary Files [file 41467_2021_24757_MOESM3_ESM.pdf]

## Description of Additional Supplementary Files

**Supplementary Movie 1. Fast camera analysis of droplet merging events in the drop-on-drop system.**

In this high-speed video, we recorded how 3 nL ADE droplets containing lysozyme crystal suspension deposited on a Kapton tape at 30 Hz are moving towards the PEI injection region with a speed of 100 mm/s. The PEI head dispenses ten ~ 60 pL droplets of the substrate (50 mg/mL aqueous GlcNAc solution) through a 100 μm orifice cartridge (big black shade on the top) at 1 kHz frequency every 30 Hz. The trigger delay time between the ADE and PEI droplet dispensing is adjusted to maximize the number of PEI drops that merge with the ADE drop. The video was recorded during XFEL beamtime at LCLS under proposal LV43 using 10 μs shutter speed and 45,000 fps frame rate. The movie is displayed at 60 fps. PEI - piezoelectric injector; ADE - acoustic droplet ejector.

**Supplementary Movie 2. Fast camera analysis of temporal and positional precision of droplets in the drop-on-drop system.**

In this high-speed video, we recorded how 3 nL ADE droplets containing lysozyme crystal suspension deposited on a Kapton tape at 30 Hz are moving towards the PEI injection region with the speed of 35 mm/s. The PEI head dispenses seven ~ 60 pL droplets of the substrate (50 mg/mL aqueous GlcNAc solution) through a 100 μm orifice cartridge (big black shade on the top) at 300 Hz frequency, every 30 Hz. The trigger delay time between the ADE and PEI droplet dispensing is adjusted to maximize the number of PEI drops that merge with the ADE drop. The video is recorded in a “synchronised” mode linked to the ADE trigger, i.e. is composed only of frames with a particular delay time after the ADE signal. The delay is set to visualise the position of the first (out of seven) PEI drops in flight. The ADE drop on the right already has the substrate added and the PEI drops that did not merge are visible on the tape. The video was recorded during XFEL beamtime at LCLS under proposal LV43 using 10 μs shutter speed and 45,000 fps frame rate. The movie is displayed at 15 fps. PEI - piezoelectric injector; ADE - acoustic droplet ejector.
