# Supplementary material for: An on-demand, drop-on-drop method for studying enzyme catalysis by serial crystallography
Source: Nat Commun. 2021 Jul 22;12:4461. doi: 10.1038/s41467-021-24757-7 (PMC8298390; doi:10.1038/s41467-021-24757-7)
Supplement: Supplementary file 6 — Reporting summary [file 41467_2021_24757_MOESM6_ESM.pdf]

nature research

Corresponding author(s): Allen M. Orville, Jan F. Kern

Last updated by author(s): Jun 22, 2021

Reporting Summary

Nature Research wishes to improve the reproducibility of the work that we publish. This form provides structure for consistency and transparency in reporting. For further information on Nature Research policies, see our Editorial Policies and the Editorial Policy Checklist.

Statistics

For all statistical analyses, confirm that the following items are present in the figure legend, table legend, main text, or Methods section.

n/a	Confirmed	
☐	☒	The exact sample size (n) for each experimental group/condition, given as a discrete number and unit of measurement
☐	☒	A statement on whether measurements were taken from distinct samples or whether the same sample was measured repeatedly
☒	☐	The statistical test(s) used AND whether they are one- or two-sided<br>*Only common tests should be described solely by name; describe more complex techniques in the Methods section.*
☒	☐	A description of all covariates tested
☒	☐	A description of any assumptions or corrections, such as tests of normality and adjustment for multiple comparisons
☐	☒	A full description of the statistical parameters including central tendency (e.g. means) or other basic estimates (e.g. regression coefficient) AND variation (e.g. standard deviation) or associated estimates of uncertainty (e.g. confidence intervals)
☒	☐	For null hypothesis testing, the test statistic (e.g. F, t, r) with confidence intervals, effect sizes, degrees of freedom and P value noted<br>*Give P values as exact values whenever suitable.*
☒	☐	For Bayesian analysis, information on the choice of priors and Markov chain Monte Carlo settings
☒	☐	For hierarchical and complex designs, identification of the appropriate level for tests and full reporting of outcomes
☒	☐	Estimates of effect sizes (e.g. Cohen's d, Pearson's r), indicating how they were calculated

Our web collection on statistics for biologists contains articles on many of the points above.

Software and code

Policy information about availability of computer code

Data collection	Open source software cctbx.xfel running DIALS 2.1 at SACLA; modified versions of Cheetah and CrystFEL at SACLA (J. Appl. Cryst. (2016). 49, 1035-1041 https://doi.org/10.1107/S1600576716005720); DIALS 2.1 at Diamond Light Source.
Data analysis	Phaser 2.8.3, Phenix 1.18.1, DIALS 2.1, Kinetiscope 1.1.1127, PyMOL 2.4.1, UCSF Chimera 1.13.1, SciPy 1.4.1, Coot 0.8.9.2, GraphPad Prism 6.0

For manuscripts utilizing custom algorithms or software that are central to the research but not yet described in published literature, software must be made available to editors and reviewers. We strongly encourage code deposition in a community repository (e.g. GitHub). See the Nature Research guidelines for submitting code & software for further information.

Data

Policy information about availability of data

All manuscripts must include a data availability statement. This statement should provide the following information, where applicable:

- Accession codes, unique identifiers, or web links for publicly available datasets
- A list of figures that have associated raw data
- A description of any restrictions on data availability

Coordinates and structure factors that were generated during the course of this study have been deposited in the Protein Data Bank with the accession codes 7BHK [http://doi.org/10.2210/pdb7BHK/pdb] (HEWL SACLA resting state), 7BHL [http://doi.org/10.2210/pdb7BHL/pdb] (HEWL SACLA 0.2 s time point), 7BHM [http://doi.org/10.2210/pdb7BHM/pdb] (HEWL SACLA 0.6 s time point), 7BHN [http://doi.org/10.2210/pdb7BHN/pdb] (HEWL SACLA 2 s time point), 7BH3 [http://doi.org/10.2210/pdb7BH3/pdb] (CTX-M-15 SACLA resting state), 7BH4 [http://doi.org/10.2210/pdb7BH4/pdb] (CTX-M-15 SACLA 0.6 s time point), 7BH5 [http://doi.org/10.2210/pdb7BH5/pdb] (CTX-M-15 SACLA 2 s time point), 7BH6 [http://doi.org/10.2210/pdb7BH6/pdb] (CTX-M-15 DLS I24 resting state) and 7BH7 [http://doi.org/10.2210/pdb7BH7/pdb] (CTX-M-15 DLS I24 10 min time point).

Protein structures used as search models in molecular replacement are accessible in the Protein Data Bank under accession codes 4ETA [http://doi.org/10.2210/pdb4eta/pdb] (HEWL) and 6QW8 [http://doi.org/10.2210/pdb6qw8/pdb] (CTX-M-15).

Information on compounds studied in this work is available in PubChem database: ertapenem (CID 23674512 [https://pubchem.ncbi.nlm.nih.gov/compound/23674512) and N-acetyl-D-glucosamine (CID 24891348 [https://pubchem.ncbi.nlm.nih.gov/substance/24891348]).

Source data for Figure 1, Supplementary Figures 5 and 6 are provided.

Field-specific reporting

Please select the one below that is the best fit for your research. If you are not sure, read the appropriate sections before making your selection.

- [x] Life sciences
- [ ] Behavioural & social sciences
- [ ] Ecological, evolutionary & environmental sciences

For a reference copy of the document with all sections, see nature.com/documents/nr-reporting-summary-flat.pdf

Life sciences study design

All studies must disclose on these points even when the disclosure is negative.

Sample size	X-ray diffraction: Number of integrated lattices (ranging between 4,974 and 18,661 lattices, depending on the dataset) was adequate to ensure minimum 10-fold multiplicity at the highest resolution shell, monotonically decreasing CC1/2 and I/σ(I) values (Nat Methods. 2017 Apr; 14(4): 443–449).<br><br>Enzymatic activity assay: Triplicates were used. This was based on similar studies using the same method (for example Biomolecules. 2020 Jun 12;10(6):899) and is a widely used procedure for this experiment type.<br><br>Fluorescence measurements: No statistical method was used to predefined the sample size. In order to determine the optimal dye concentration a concentration series was ejected at various tape speeds. Number of repetitions was a compromise between experimental feasibility and ensuring that the number of replicates is sufficient to obtain reliable results. We determined that 10-50 repetitions was satisfactory.
Data exclusions	No data were excluded from the study.
Replication	In fluorescence measurements, each data point represents the average of four scans integrated over 300 ms repeated between 10 and 50 times. In enzymatic activity assays, the values represent the average of three repeats.
Randomization	No experimental group allocation was carried out and so randomization is not relevant to the methods used in this study.
Blinding	No experimental group allocation was carried out and so blinding is not relevant to the methods used in this study.

Reporting for specific materials, systems and methods

We require information from authors about some types of materials, experimental systems and methods used in many studies. Here, indicate whether each material, system or method listed is relevant to your study. If you are not sure if a list item applies to your research, read the appropriate section before selecting a response.

Materials & experimental systems

n/a	Involved in the study
x	☐ Antibodies
x	☐ Eukaryotic cell lines
x	☐ Palaeontology and archaeology
x	☐ Animals and other organisms
x	☐ Human research participants
x	☐ Clinical data
x	☐ Dual use research of concern

Methods

n/a	Involved in the study
x	☐ ChIP-seq
x	☐ Flow cytometry
x	☐ MRI-based neuroimaging
