# Supplementary material for: An on-demand, drop-on-drop method for studying enzyme catalysis by serial crystallography
Source: Nat Commun. 2021 Jul 22;12:4461. doi: 10.1038/s41467-021-24757-7 (PMC8298390; doi:10.1038/s41467-021-24757-7)
Supplement: Supplementary file 7 — Source Data [file 41467_2021_24757_MOESM7_ESM.zip › Explanations.docx]

Fluorescence & Simulation Data

# Overview

The data is sorted in the following order:

1. **Raw Data**For completeness, the folder contains all data including the applied background, and the fluorescence of the dyes with the mixing turned on and off.
   The parameter space can be found below.
2. **Averaged Spectra**
   This folder contains the averaged spectra obtained from the raw data for each set of parameters with the mixing turned on and off.
   The parameter space can be found below.
3. **Fit spectra**
   Contains the normalized spectra of the dyes and the diode used for interpolation and fitting of the data (Supplementary Figure 5a). How these spectra are obtained is described in the methods section.
4. **Fit results**
   Contains the unnormalized fluorescence components obtained from the fit of the fit spectra onto the averaged spectra (Figure 1).
   Also the exponential fit is given. The fitted curve is used to normalize the data to the maximum value.
5. **Simulation data**
   Contains the results from the simulations
   Figure1 diffusion: ‘concentration_time-diffusion’; collision: ‘Bidirectional-mixing-210111’ columns 4 and 5
   Supplementary Figure 6: ‘Jet_vs_nonjet_graph’

# Parameter-space of saved data

| File nb | 9 | 7 | 5 | 12 | 11 | 10 | 6 | 8 |
| --- | --- | --- | --- | --- | --- | --- | --- | --- |
| Tape speed [mm/s] | 300 | 200 | 100 | 100 | 100 | 100 | 50 |  |
| Interaction time until detection | 0.23 | 0.35 | 0.7 | 0.7 | 0.7 | 0.7 | 1.4 |  |
| Droplet ratio estimate | 0.9 | 1.35 | 2.7 | 0.27 | 0.68 | 1.35 | 5.4 |  |
| Frequency Poly Pico [kHz] | 1 | 1 | 1 | 0.1 | 0.25 | 0.5 | 1 |  |
| Normalized inverse tape speed | 0.33 | 0.5 | 1 | 1 | 1 | 1 | 2 |  |
| Used in rise (fura/fluo) | x / x | x / x | x |  |  | x / x | x / x |  |
| *comment* |  |  |  | *Not hitting* | *Not hitting* |  |  | *control* |
